# Mass spectrometry transanal minimally invasive surgery (MS-TAMIS) to promote organ preservation in rectal cancer

**DOI:** 10.1007/s00464-019-07140-y

**Published:** 2019-10-15

**Authors:** Sam Mason, Eftychios Manoli, Liam Poynter, James Alexander, Petra Paizs, Afeez Adebesin, Robert Goldin, Ara Darzi, Zoltan Takats, James Kinross

**Affiliations:** 1 Department of Surgery and Cancer, Imperial College London, St Mary’s Hospital, 10th Floor QEQM Building, Paddington, London W2 1NY UK; 2 Department of Histopathology, Imperial College London, South Kensington, UK

**Keywords:** Mass spectrometry, TAMIS, Organ preservation

## Abstract

**Background:**

Transanal minimally invasive surgery (TAMIS) is deployed for organ preservation in early rectal cancer and significant rectal polyps. Rapid evaporative ionisation mass spectrometry (REIMS) provides biochemical tissue analysis, which could be applied intraoperatively to give real-time tissue feedback to the surgeon and decrease the risk of an involved margin. However, the accuracy and feasibility of this approach have not been established.

**Methods:**

In this prospective observational study, patients undergoing resection of rectal adenomas or carcinomas were recruited. An electrosurgical handpiece analysed tissues ex vivo using diathermy, with the aerosol aspirated into a Xevo G2-S ToF mass spectrometer. The relative abundance of lipids underwent predictive statistical modelling and leave-one-patient-out cross-validation. The outcomes of interest were the ability of REIMS to differentiate normal, adenomatous and cancerous tissue, or any disease subtype from normal. REIMS was coupled with TAMIS for in vivo sampling, assessing the accuracy of tissue recognition and distinguishing bowel wall layers.

**Results:**

Forty-seven patients were included, yielding 266 spectra (121 normal, 109 tumour and 36 adenoma). REIMS differentiates normal, adenomatous and cancerous rectal tissues with 86.8% accuracy, and normal and adenomatous tissue with 92.4% accuracy and 91.4% accuracy when differentiating disease from normal. We have performed the first five in-man mass spectrometry augmented TAMIS (MS-TAMIS). In real time, MS-TAMIS can differentiate rectal mucosa and submucosa based on their relative abundance of triglycerides and glycerophospholipids. The ex vivo accuracy distinguishing diseased and normal tissues is maintained in vivo at 90%, with negative predictive value of 95%. The system identified a deep and lateral involved tumour margin during TAMIS.

**Conclusions:**

REIMS distinguishes rectal tissue types based on underlying lipid biology, and this can be translated in vivo by coupling it to TAMIS. There is a role for this technology in improving the efficacy of resection of early rectal cancers.

Early rectal cancers and significant rectal polyps are most commonly treated using a direct-to-TME surgery approach, with increasing use of local excision on platforms such as transanal minimally invasive surgery (TAMIS). Local excision benefits from lower morbidity, mortality, length of hospital stay and cost compared to radical surgery [1]; however, there is a fear of oncological non-inferiority.

A key factor in oncological outcome following local excision is the completeness of resection. First, this requires accurate preoperative risk stratification to ensure only patients whose lesions are suitable undergo local excision, considering pathological status (presence of high-risk features such as invasion depth and differentiation status [2]) and technical factors (size, shape and location of the lesion). The second requirement to ensure satisfactory oncological outcome is the ability of the surgeon to remove the lesion en bloc with no involved margins, despite it being technically feasible [3]. The challenge here is that neoplastic disease at the deep at lateral margins can be microscopic, such that the surgeon is unable to identify it through visual or haptic feedback. No tools currently exist which can give real-time tissue feedback to decrease the risk of involved surgical margins.

In this study, we present an augmentation to the TAMIS platform by linking it to our previously described rapid evaporative ionisation mass spectrometry (REIMS) method, which provides real-time analysis of lipid molecules present in the diathermy plume generated during electrosurgical tissue dissection [4–6]. This pilot study describes the ex vivo validation of REIMS for tissue recognition, followed by first-in-man augmentation of TAMIS with mass spectrometry.

## Materials and methods

This was a prospective observational cohort study of adults undergoing an elective resection of a rectal carcinoma or adenoma either using a surgical or endoscopic approach at two hospitals between November 2014 and April 2019. The study had ethical approval from the London Research Ethics Committee (14/EE/0024). Patients were identified preoperatively, and all gave written and informed consent. Exclusion criteria were whether the patient suffered from inflammatory bowel disease, a polyposis syndrome, had a tumour histological subtype that was not adenocarcinoma or if the consent was withdrawn.

The study was conducted in two phases: first, an ex vivo analysis of diagnostic accuracy and second, an in vivo augmentation of TAMIS using REIMS.

### Phase 1—ex vivo REIMS diagnostic accuracy

Upon excision of the surgical specimen, approximately 500 mg of macroscopically tumour and normal associated mucosa (sampled at 10 cm) was sampled ex vivo under supervision of a histopathologist. This was bio-banked at −80 °C until future REIMS analysis.

REIMS analysis was conducted on 50–100 mg of freshly thawed tissue using a modified electrosurgical handpiece applying monopolar cutting diathermy at a power of 15 W. The handpiece modification was such that any aerosol created during the application of electrocautery was immediately aspirated through PTFE tubing and directly into a Xevo G2S QToF mass spectrometer via a Venturi interface (Waters Corporation, USA). The mass spectrometer was optimised to measure the relative abundance of negatively charged ions, with the mass/charge ratio (m/z) of 600–1000 used for statistical analysis given that this is the range that includes the glycerophospholipids and triglycerides of interest. Following REIMS analysis, the tissue was formalin-fixed, paraffin-embedded and underwent haematoxylin and eosin staining. The tissue subtype was diagnosed by an expert gastrointestinal histopathologist following the guidelines of the Royal College of Pathologists [7].

All raw spectral data underwent preprocessing including total ion count normalisation and log transformation of the signal intensity. Visual assessment of each spectrum was conducted using Abstract Model Builder (proprietary to Waters Corporation, USA), and signal intensity-to-background noise ratio was assessed. Spectra were excluded from analyses if they there was insufficient signal intensity-to-background noise ratio (defined as the median intensity of the greatest 20 peaks divided by the median overall intensity equalling less than 1500), the presence of contamination or less than 20% target tissue of interest in the histological validation section.

Initial exploratory analysis and feature reduction were performed using principle component analysis (PCA), an unsupervised approach which orthogonally transforms data to reduce the dimensionality into components over which the maximum variation exists. Modelling was then conducted using orthogonal partial least squares discriminant analysis (OPLS-DA), a supervised regression method where dependent variables (such as tissue type) are differentiated using the multivariate spectral data. Cross-validation was conducted using a leave-one-patient-out approach, where an OPLS-DA model is created using the data from all but one patient, and then, classification predictions for this patient are made by using the Mahalanobis distance to each class. Once conducted for each patient, a contingency table could be created to determine metrics such as overall diagnostic accuracy, sensitivity and specificity. ANOVA was conducted in R Studio to identify the masses of ions which were responsible for class discrimination, using Benjamini *p* value correction and defining a cut-off of *p *≤ 0.05. Metabolite identification was performed using reference databases and tandem mass spectrometry (ions of interest are fragmented, and their structure is identified using these fragments.) It was not possible to perform a reliable power calculation during the design of the study due to challenges with such a multivariate data type and a lack of pilot data.

The primary outcome was the diagnostic accuracy of REIMS in the differentiation of rectal cancer, adenoma and normal tissue. The secondary outcome was the ability of REIMS to predict the presence of disease (either adenoma or cancer) in rectal tissue.

### Phase 2—in vivo MS-TAMIS

Between August 2018 and June 2019, patients with significant rectal polyps and early rectal cancers were all reviewed in a multidisciplinary team meeting, and if local surgical excision was indicated, the patient was recruited for this phase of the study.

**Fig. 1:**
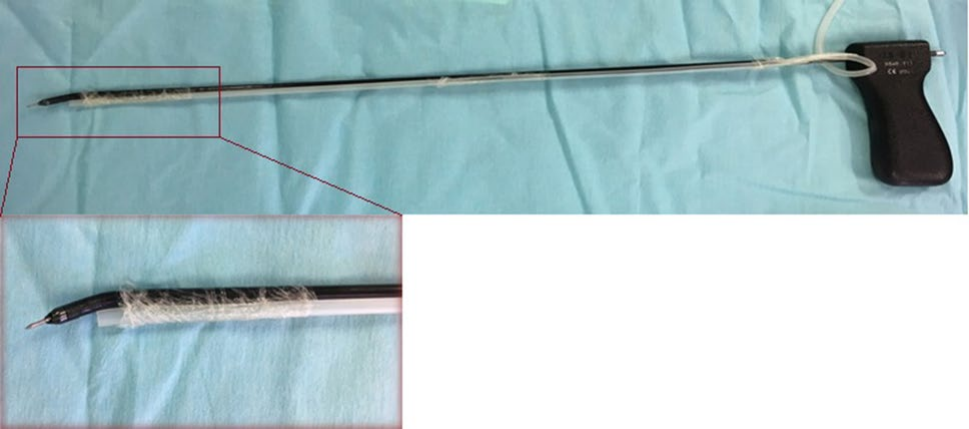
Aerosol aspiration tubing taped alongside dissecting instrument for TAMIS. A constant negative pressure automatically aspirates the aerosol during dissection

Prior to starting the TAMIS procedure, a sterile PTFE tube was taped alongside the dissection instrument, which was used for continuous aspiration of the electrocautery aerosol into the mass spectrometer (Fig. 1). Diathermy used during TAMIS was in the coagulation mode at 25 W. In lesions of over 20 mm, the luminal aspect of the polyp was sampled using a single 0.5 s diathermy application and data were collected throughout the entire procedure. Video sequences of the procedure were captured using GoPro cameras or the formal Storz system, to allow correlation between raw spectra and tissue being analysed. All tissue samples were submitted for formal histopathological assessment in keeping with standard of care, with involved tumour margins defined as neoplastic disease within 1 mm of the cut edge.

REIMS analysis of the electrocautery aerosol during TAMIS and the preprocessing of spectral data was identical to the methodology used in phase 1; however, signal-to-noise ratio exclusion was not applied due to differences in signal between ex vivo and in vivo systems.

The statistical model differentiating diseased (tumour or adenoma) and normal tissues from phase 1 of the study was uploaded into AMX Recognition software (Waters corporation, USA), which makes predictions on the tissue type of new spectra it is exposed to by referencing the uploaded spectral library. This can be conducted in real time, and it is possible to feedback the results using a visual user interface. The predictions were compared to the histological subtype at the point of dissection, which is identified through a combination of co-registering the mass spectra to the procedural videos and examining the detailed histological report. This allows creation of a 2 × 2 misclassification table and calculation of standard diagnostic accuracy metrics. The primary outcome of this in vivo study phase was the feasibility and safety of REIMS augmentation of TAMIS. Secondary outcomes were the ability of REIMS to differentiate bowel wall layers during dissection and the accuracy of REIMS in differentiating diseased and non-diseased rectal tissues.

## Results

### Phase 1—ex vivo REIMS diagnostic accuracy

Fifty-three patients were recruited for inclusion in the ex vivo study phase, all of whom had tissue sampling and analysis with REIMS. A total of six patients were excluded: five due to insufficient spectral quality (recognised by a poor signal intensity-to-background noise ratio) and one case where there was no tumour tissue in the research specimen.

The demographic and pathological data of the 47 patients included are given in Table 1. The majority underwent radical surgical resection (77%) with the remaining having endoscopic or local surgical excision (17% and 6%, respectively). Thirty adenocarcinomas were analysed; however, one of these patients did not have a radical excision, and therefore, full histological assessment could not be conducted.

**Table 1:** Demographic and pathological data on the 47 patients included in the ex vivo study

Demographic	Number
Mean age (range)	70 (36–87)
Gender (M:F)	25:22
Ethnicity	
Caucasian	32
African	5
Asian	4
Unknown/other	6

Pathology of sampled tissue	Number
Adenocarcinoma	30
Adenoma	12
Normal mucosa	35

Histology of carcinoma^a^	Number
Tumour stage	
T1/2	12
T3/4	17
Nodal stage	
N0	18
N1/2	11
Use of neoadjuvant therapy	
Absent	26
Present	3

^a^Twenty-nine of the 30 tumour patients underwent radical resection allowing full histological staging

In total, 266 raw spectra were included following REIMS analysis of the 77 tissue samples included in the analysis (109 tumour, 36 adenoma and 121 normal). PCA demonstrated that there was no evidence of clustering based on gender, age or resection type (data not shown).

**Fig. 2:**
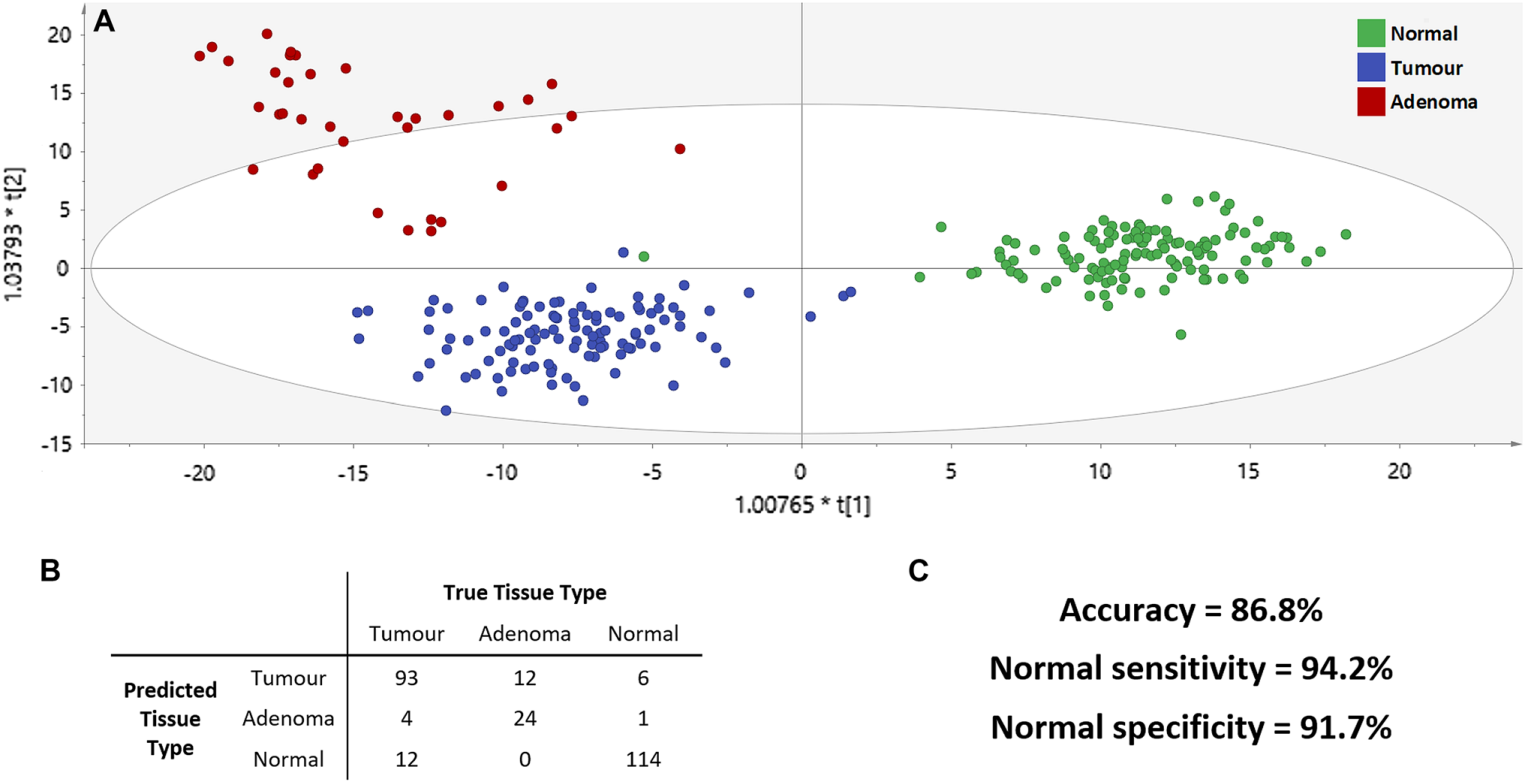
OPLS-DA plot of REIMS in the differentiation of rectal tissue type (**A**) with the contingency table following cross-validation (**B**) and accuracy metrics (**C**)

REIMS had an overall diagnostic accuracy of differentiating cancer, adenoma and normal tissue of 86.8% (Fig. 2). Incorrect classifications were largely due to adenoma tissue being predicted as tumour (33.3%) or tumour tissue predicted to be normal (11%). When tissue is classified as ‘disease’ (tumour or adenoma) or ‘no disease’ (normal), REIMS has an accuracy of 91.4%. The sensitivity, specificity, PPV and NPV for diseased tissue were 89.7%, 93.4%, 94.2% and 88.3%, respectively. The false negative rate of diseased samples was 10.3%, with no cause identified when comparing technical parameters, clinical metadata and PCA clustering between spectra which classified correctly and incorrectly. When comparing normal and adenomatous tissue, REIMS had an accuracy of 92.4%.

**Fig. 3:**
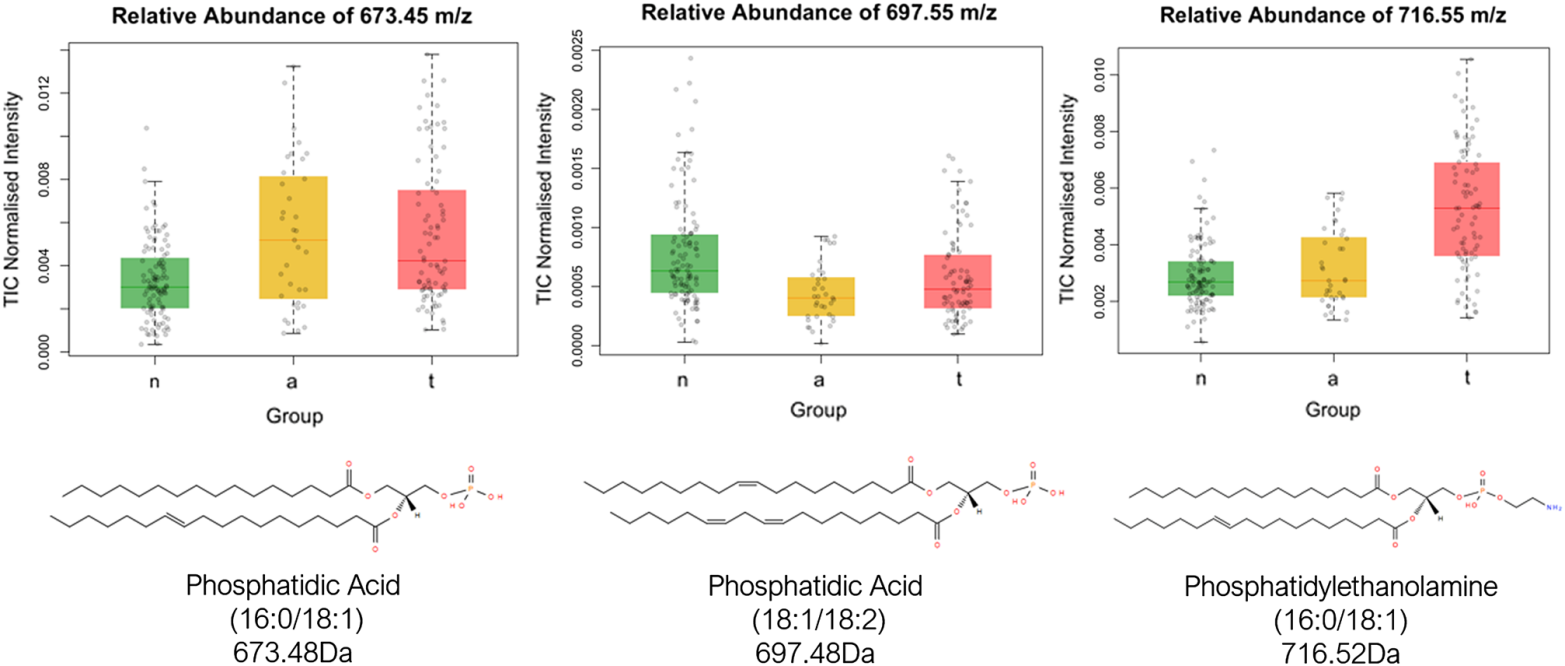
Box plots of the differential abundance of three lipids across rectal normal mucosa, adenoma and tumour tissue. The structure of them all was identified using tandem mass spectrometry, and the true deprotonated mass is given

The mass of the lipid metabolites responsible for the differentiation between tissue types was identified, with the structure of three shown in Fig. 3. It is not possible to make general statements about classes of lipids which are increased or decreased in abundance within different tissue types without identifying the structure of a large majority of significant ions in the predictive model.

### Phase 2—in vivo MS-TAMIS

Five patients underwent MS-TAMIS and were included in the study. Three had early rectal adenocarcinomas with no evidence of nodal disease on preoperative MRI, and two had advanced adenomas with no invasive malignancy.

**Fig. 4:**
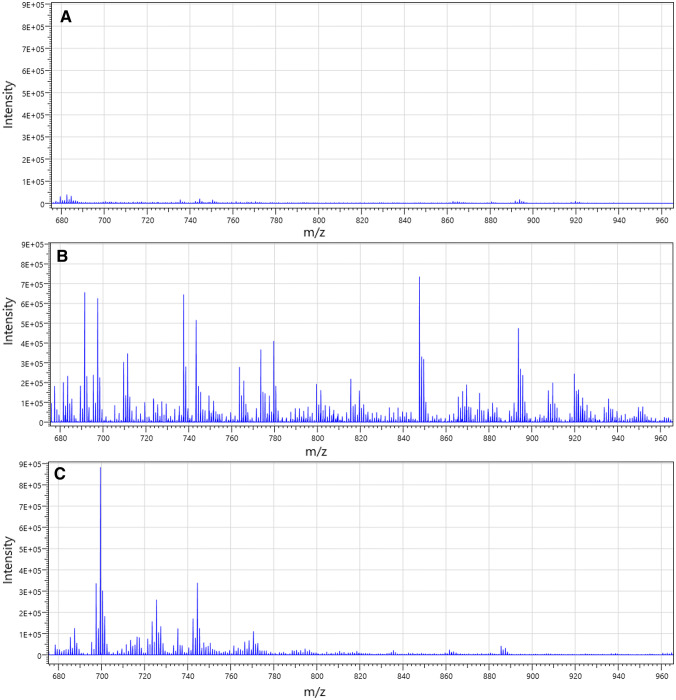
Representative spectra collected in vivo prior to protocol optimisation (**A**), in vivo after protocol optimisation (**B**) and ex vivo (**C**), across the mass–charge range of 680–960. It is apparent that following optimisation, the lipid ion signal intensity in vivo can reach those levels seen ex vivo

The first four MS-TAMIS cases were used for staff familiarisation and method optimisation, where technical parameters were manipulated to increase the quality of the spectral data collected. This included moving from a Venturi interface to direct aerosol aspiration, developing techniques of removing contamination in the aspiration tube intraoperatively and adjusting the flow rate of the co-aspirated solvent isopropanol. Spectra were collected during dissection with it being noted that total ion intensity increased throughout the optimisation process, as demonstrated in representative spectra in Fig. 4. As the focus of this process was to increase spectral quality, no clinical annotation was performed, and therefore, tissue recognition analysis was not conducted. Furthermore, no patient safety concerns were identified when using REIMS coupled to the TAMIS platform.

**Fig. 5:**
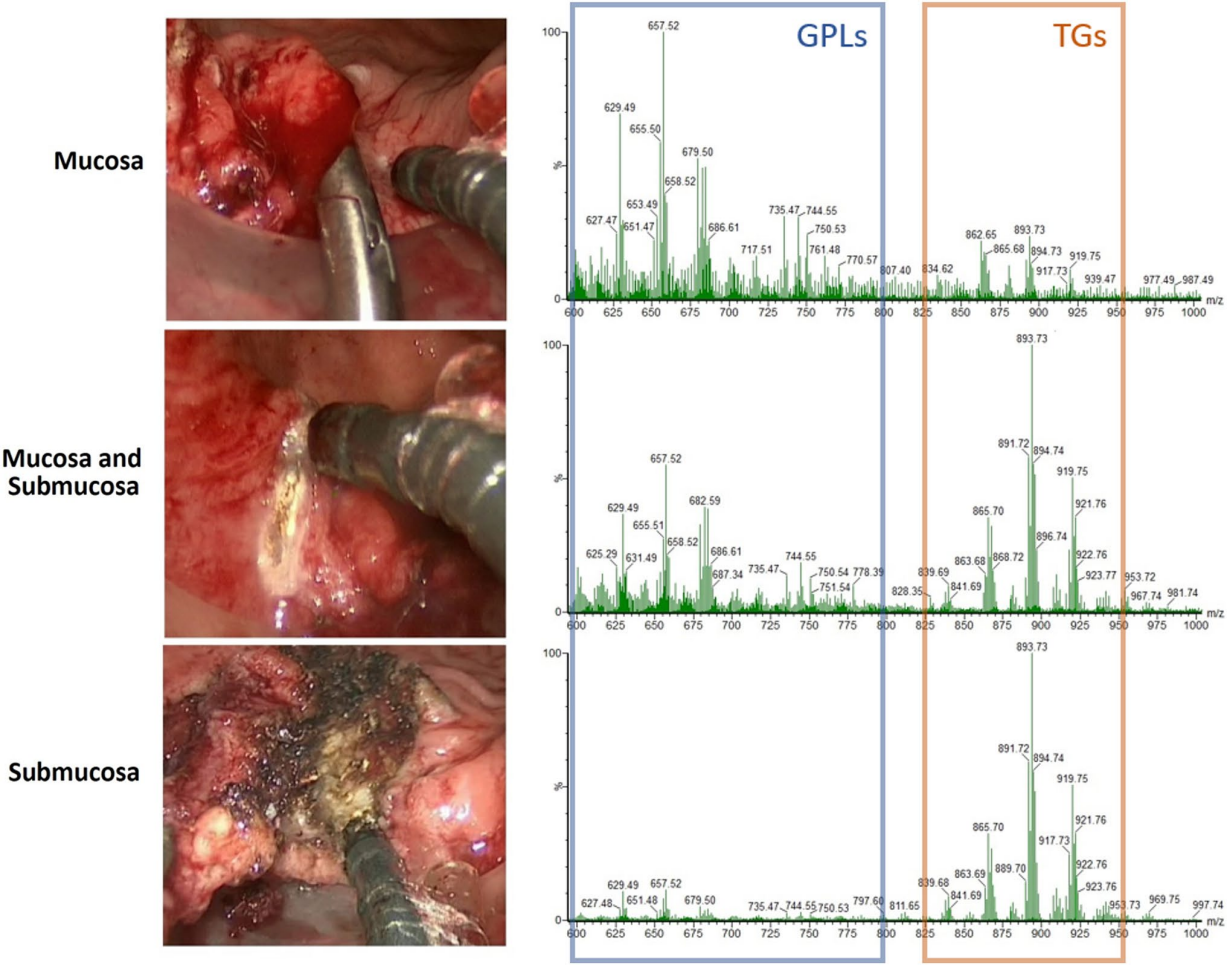
Representative spectra from dissection of the mucosa, a combination of mucosa and submucosa, and solely submucosa. The ratio of the glycerophospholipids (blue) to the triglycerides (orange) falls as the dissection moved from mucosa to the submucosa (Color figure online)

REIMS was able to distinguish the layers of bowel wall during TAMIS dissection using the distinctive lipid composition at each layer, particularly the ratio between glycerophospholipids (GPLs) and triglycerides (TGs). As shown in Fig. 5, MS-TAMIS could identify the mucosa (typified by relative richness in GPLs), when a mixture of both mucosa and submucosa was being dissected (both GPLs and TGs present) and when solely submucosa was being dissected (relative richness of TGs). No representative spectra were collected for the muscularis layer as this was not dissected out during the cases. This validates previous work by our group where the distinctive lipid features of the mucosa and submucosa were identified using mass spectrometry imaging techniques (data as yet unpublished).

After method optimisation was complete, MS-TAMIS was prospectively applied, where it was possible to co-register the mass spectra generated at every point of dissection with a high-definition video of the procedure. The case was of a 68-year-old man with a 15 mm rectal lesion, 48 mm from the anal verge, preoperatively staged as T1 on MRI. Histological assessment of the specimen following MS-TAMIS demonstrated a T2 poorly differentiated adenocarcinoma, with involved margins both deep (0.8 mm from cut edge) and laterally at the 7–8 o’clock position as per the view of the surgeon (with tumour at the cut edge). Recognition software was applied to the 100 discrete burns of the dissection, and REIMS was able to identify both involved margins, with an overall accuracy of 90%. The negative predicted value for tumour was 95%, with four cases of a spectrum being predicted to be normal despite being in diseased tissue.

## Discussion

This was a prospective observational cohort study, demonstrating that the analysis of cellular lipids using REIMS can differentiate rectal tissues in real time. In addition, it has been demonstrated that it is feasible to augment TAMIS with REIMS to potentially provide the surgeon with accurate and meaningful clinical data.

The diagnostic potential of REIMS is dependent on the hypothesis that different tissue phenotypes have a distinct composition of lipid species in the cells, as the technique is directly analysing the underlying tissue chemistry. Altered and dysregulated lipid metabolism has been well documented in colorectal cancer. This extends beyond the increased expression of fatty acid synthase to meet increased energy demands and the need for lipid bilayers, to an ever-increasing understanding of the role of glycerophospholipids in tumour viability, proliferation and metastasis [8, 9]. How changes at the genomic, transcriptomic and proteomic levels directly affect the molecular makeup of tissue is poorly understood, but the relationship between tissue phenotype and lipid composition appears to have been confirmed based on these and past results [5]. The benefit of this is that the exact metabolic differences between classes can be described at a molecular level to give an insight into underlying biological processes. This study starts to draw the link between biology and clinical phenotype, to attempt to make inferences relevant to clinical care intraoperatively.

Local excision of an early rectal cancer using a technique such as TAMIS protects patients against the morbidity and mortality associated with radical surgery including prolonged hospitalisation, anastomotic leak, anterior resection syndrome and sexual dysfunction. However, this is only in the best interests of the patient if oncologic outcomes are non-inferior and it is likely that concerns over this are partly responsible for the underutilisation of TAMIS [10]. The fact that REIMS can accurately differentiate rectal normal mucosa, adenoma and cancer in real time indicates the potential to augment platforms for local surgical excision by providing real-time lateral and deep margin detection. The largest source of inaccuracy in the model was that 33% of rectal adenomas were predicted to be tumours, which, although histologically incorrect, does make biological sense as many of the metabolic and genomic changes seen in colorectal cancer begin to be evident at the adenoma stage [11]. This is largely resolved when using a more clinically relevant model of ‘disease’ (tumour or adenoma) or ‘no disease’ (normal), increasing the accuracy to 91.4%. Being able to make such distinctions is likely to improve oncological outcomes by allowing the surgeon to modify the dissection strategy intraoperatively to promote an R0 resection. The 88.3% NPV of diseased tissue (where histologically diseased tissue is recognised as normal) could have clinical impact, as it risks not dissecting the entirety of the lesion. It was found that in all but two of the samples where there were such misclassifications, other REIMS spectra correctly identified the tumour, in which case the surgeon could be alerted to the presence of high-risk tissue and the dissection strategy is adjusted. Using this definition (where any spectra from a sample recognise tumour) raises the NPV to 97.4%.

Phase 2 of this study demonstrates that it is feasible to translate the ex vivo potential of REIMS into the operating room by coupling it to the diathermy used during TAMIS, a world first. Raw spectra could be collected using this technique without interference to the surgeon or affecting the operating time. Further optimisation is required to increase the quality of the spectra generated and overcome technical issues such as a long length of aspiration tube and aspiration tube blockage from blood; despite this, we can still collect meaningful clinical data. The abundance of triglycerides in the submucosa and glycerophospholipids in the mucosa is such that a ratio between the two can be used to identify the layers being dissected. This is clinically relevant as feeding this back to the surgeon in real time could improve the quality of dissection. Furthermore, the ex vivo tissue recognition accuracy of REIMS was replicated when applied in vivo during MS-TAMIS, with correct prediction of two involved tumour margins (lateral and deep). The surgical precision that this provides does not exist with current techniques. We have not allowed surgeons to be aware of real-time analysis due to ethical considerations, but this will be the subject of future prospective work. Using REIMS in vivo certainly has logistical and technical challenges which are currently an obstacle to routine clinical implementation, such as a large machine size, technical expertise required for operation and constant requirement for electrical power. The technology will need to continue to progress to address such issues.

This work has several limitations. Analyses from four patients (7.5%) had to be excluded from the ex vivo phase of the study due to poor signal intensity. This failure rate was addressed through modulation of technical parameters (flow rate of co-aspirated solvent), and as such, no further exclusions were necessary in the final 40 patients. Poor signal intensity is a risk to REIMS analysis; however, it appears that optimisation and standardisation of technical parameters act sufficiently to mediate this risk. We were not able to draw a causal link between altered lipid metabolism, differential cellular lipid composition and clinical phenotype; however, the statistical associations in this metabolomic analysis are strong. REIMS biologically samples a small portion of a lesion to make predictions; however, it is established that there can be significant intratumour heterogeneity. It may be possible that a clonal population can exhibit different metabolic profiles despite having the same histological diagnosis, which could be a source of misclassification in the statistical modelling. Sampling a lesion in multiple locations raises the issue of how to deal with potentially contradictory predictions; however, as discussed, this could be managed by recognising a lesion as malignant if any of the analyses predict this histological subtype. The ex vivo portion of this study included 77 tissue samples from 47 patients, and therefore, there is a risk that this work is underpowered, especially given that the most recent analytical batch of 10 extra patients increased accuracies by 5% (data not shown). Performing power calculations using metabolic data with high dimensionality is very challenging. All metabolic data risk overfitting of statistical models; however, the leave-one-patient-out cross-validation technique used in this manuscript is a robust method to mitigate against this. Prospective testing of this technology in larger patient cohorts is necessary.

We conclude that chemical analysis of lipids within rectal tissues using REIMS appears to allow tissue differentiation and risk stratification of rectal cancers. Furthermore, this can be translated into the operating theatre to potentially provide the surgeon with meaningful clinical data to improve the quality of rectal cancer excision.
